# A survey of metal profiles in some traditional alcoholic beverages in Nigeria

**DOI:** 10.1002/fsn3.163

**Published:** 2014-09-09

**Authors:** Chukwujindu M A Iwegbue, Anwuli L Ojelum, Francisca I Bassey

**Affiliations:** 1 Metals and Trace Organic Research Group, Department of Chemistry, Delta State UniversityP.M.B 1, Abraka, Nigeria; 2 School of Chemistry and Physics, University of KwaZulu-NatalWestville Campus, Private Bag X54001, Durban, South Africa; 3 Department of Chemistry, University of CalabarCalabar, Nigeria

**Keywords:** Dietary intake, essential metals, Nigeria, target hazard quotients, toxic metals, traditional alcoholic beverage

## Abstract

The concentrations of Cd, Pb, Ni, Cr, Cu, Co, Fe, Mn, Zn, Mg, Ca, K, and Na were determined in some traditional alcoholic beverages (oil palm wine, raphia palm wine, burukutu, pito, ogogoro) consumed in southern Nigeria, with a view to providing information on the dietary intakes of essential metals and exposure of humans to toxic metals. The concentrations of these 13 elements were determined by atomic spectrometry after nitric acid/hydrogen peroxide digestion. The mean concentrations of the metals (mg/L) in the samples ranged from 0.02 to 0.05 for Cd; 0.01 to 0.19 for Pb; nd to 0.11 for Ni, nd to 0.15 for Cr; 0.09 to 0.60 for Cu; 0.01–0.08 for Co; 0.30 to 10.3 for Fe; 0.02 to 3.97 for Mn; 0.12 to 3.84 for Zn; 2.08 to 301.3 for Mg; 2.21 to 49.2 for Ca; 35.05 to 926.1 for K; 6.30–58.1 for Na. The mean concentrations of metals in these alcoholic beverages were below statutory limits for the metals in alcoholic beverages and were similar to concentrations found in other alcoholic beverages in the literature. The estimated daily intakes of metals from the consumption of these alcoholic beverages were less than 2% of the recommended dietary allowance values except for Cd and Pb. The individual and combined metals target hazard quotient values were less than 1 except for raphia palm wine and burukutu. From the estimated target hazard, no long life health concerns of metals are associated with the consumption of these alcoholic beverages.

## Introduction

The determination of metals in environmental, biological, and food samples has attracted significant attention due to their double-edged roles which range from the nutritional requirements of essential elements to the toxicity effects associated with the overload of these elements or their compounds (Hague et al. 2008; Mihaela et al. 2009). The knowledge of metals in foods is essential for calculating the dietary intakes of essential metals and evaluation of human exposure to toxic elements (Iwegbue 2010).

Some metals such as Cu, Fe, Mn, Zn, Ca, Mg, K, and Na are essential. For instance, Fe and Cu ions are required for the synthesis of metalloproteins (Naughton and Petroczi 2008a). However, excess intakes of trace metal ions have been implicated in pathological events such as the deposition of iron oxides in Parkinson's disease (Power et al. 2003). In addition to aiding the neurological depositions, these redox active metal ions also enhance premature aging (Naughton and Petroczi 2008a,b)and cause oxidative damage, a key component of chronic inflammatory disease and a suggested initiator of cancer (Valko et al. 2006; Naughton and Petroczi 2008a).

Toxic metals, such as Cd and Hg, are known to impair kidney functions and reproductive capacity, cause hypertension, tumor, and hepatic dysfunction, whereas Pb can cause renal failure, liver damage, impaired hearing, or cause mental retardation, while elevated levels in women may result to a shortened gestation period (Iwegbue 2010).

Palm wine, burukutu, pito, and local gin (ogogoro or kai-kai) are traditional alcoholic beverages consumed by more than 10 million people in West Africa (FAO 1998; Ukhun et al. 2005). Palm wine is a sweet effervescent drink obtained from sap of the oil palm (*Elaeis guineense*) and raphia palm (*Raphia hookeri*). Palm wine is a rich medium containing sugar, protein, amino acids, alcohol, and mineral (Ezeagu et al. 2003). Burukutu is a vinegar-like flavored alcoholic beverage prepared from steeping sorghum grains in water overnight, following which excessive water is drained. The grains are then spread out onto a mat or tray, covered with banana leaves and allowed to germinate. During germination process, the grains are watered on alternate days and turned over at intervals. Germination continues for 4–5 days, following which the dried malt is ground into a powder. Gari (a farinaceous fermented cassava product) is added to a mixture of ground malt and water in a ratio of one part gari to two parts malt and six parts water. The resulting mixture is allowed to ferment for 2 days, boiled for approximately 4 h and allowed to mature for 2 days. The resulted product is a cloudy alcoholic beverage. The alcohol contents of burukutu range from 3% to 6%. Pito is prepared by shaking cereal grain (maize, sorghum, or a combination of both) in water for 2 days, followed by malting, and allowing them to sit for 5 days in baskets lined with moistened banana leaves. The malted grains are ground, mixed with water, and boiled. The filtrate thus retained is allowed overnight or until it assumes a slightly sour flavor. This is further boiled to a concentrate. A starter from the previous brew is added to the cooled concentrate and allowed to ferment overnight. The product obtained is a dark brown liquid which varies in taste from sweet to bitter. It has an alcohol content of 3%. The local gin (ogogoro) is produced from the distillation of fermented oil palm wine or raphia palm wine, and its percentage alcohol by volume varies from 40% to 60% depending on the source.

The presence of metals in foods and beverages, except for accidental or criminal action reflects the environment. Water is the main component of beverages; as a consequence, the concentrations of metals in beverages may be related to the purity of the water of used in the production processes. The processing of these traditional alcoholic beverages is carried out as a small-scale enterprise involving the possible use of low water quality, there is a distinct possibility of contamination of the products with undesirable elements. These alcoholic beverages (palm wine, burukutu, pito, and local gin) are locally produced, readily available, cheap, and are mostly consumed by low-income earners in preference to the more expensive brewed beer, spirit, and rums, etc. However, the consumption of these drink types is not restricted to low-income earners alone, higher income earners also consume these drinks. These drink types are the favorite drinks for traditional occasions in most West African countries. Despite the large consumption of palm wine, burukutu, pito, and ogogoro, their inorganic profile remained undefined. The processing of palm wine essentially involves the filtration of fresh palm wine, dilution with water, bottling, and pasteurization (Ukhun et al. 2005). Palm wine is essentially stored in plastic kegs, glass jars and calabash. One of the most frequent complaints of palm wine consumers is adulteration of the product by using water and artificial sweetness, which sometimes result in diarrhea, abdominal pains, and other stomach problems (Ukhun et al. 2005).

A survey of the literature revealed that there are few studies on metal contents of these alcoholic drinks and these studies are limited in the scope of the elements and drink types surveyed. However, no study has been reported on the daily intakes of metals and the combined effects of frequent ingestion of multiple metal ions, which can be addressed as a function of the quantified level of concerns in the form of Target Hazard Quotient (THQ) values arising from the consumption of these alcoholic drinks. The objective of the present study was to evaluate the concentrations of metals in these traditional alcoholic beverages with a view to providing information on the lifelong effects of these metals on humans arising from consumption of these drinks.

## Materials and Methods

### Sample collection

Samples of oil palm and raffia palm wine were collected from different wine tappers in widely spread locations in southern Nigeria, while local gin (ogogoro) was collected from local distilleries in Delta and Bayelsa States of Nigeria. Burukutu and Pito samples were obtained from the producers from the southwestern Nigeria. The samples were conveyed to the laboratory in a clean, nitric acid-treated plastic cans and are freezed until required for analysis. All collected samples were freshly produced.

### Reagents

All reagents used were of analytical grade. Working standards of Cd, Pb, Ni, Cr, Cu, Co, Fe, Mn, Zn, Ca, Mg, K, and Na were prepared by diluting concentrated stock solution (Merck, Darmstadt, Germany) of 1000 mg/L with 0.25 mol/L nitric acid.

### Sample preparation

Prior to analysis, palm wine samples are degassed using ultrasonic bath for 5 min. A 20 mL of aliquot of the sample was mixed with 2 mL of nitric acid and 2 mL of hydrogen peroxide in a digestion tube. The mixture was heated for 1 h (100°C) until complete clarification. The sample was allowed to cool to room temperature, filtered, and diluted to 25 mL with 0.25 mol/L nitric acid. Analytical blanks were prepared in a similar manner, but omitting the test sample.

### Chemical analysis

All digested samples were analyzed in triplicate using flame atomic absorption spectrophotometry (Analyst 200; Perkin Elmer, Norwalk, CT). Sodium and potassium were analyzed in the samples using flame photometer. Blanks and calibration standard solution were analyzed in the same way as the samples.

### Quality control and statistical analysis

Appropriate quality assurance procedure and precautions were carried out to ensure reliability of the results. Samples were carefully handled to avoid contamination. Glassware and sample containers were soaked in 1 mol/L HNO_3_ for 48 h and rinsed with ultrapure water. In absence of a certified reference standard, a recovery test of the total procedure was carried out for the metals by spiking analyzed samples with aliquots of the metal standards and then reanalyzing the samples. The results of the recovery studies for the various metals were greater than 95%. In addition to spike recovery studies, interlaboratory comparison of results was carried out by analyzing 10% of total sample in an independent analytical laboratory (Dukoria Nig. Ltd., Warri, Nigeria). The results of interlaboratory studies showed a strong agreement. Procedural reagent blank test was carried out over the entire procedure using the same amounts of reagents (including water) used in analysis, omitting only the sample. The procedural blank was used to correct calculated sample concentrations for contamination from reagents and procedural manipulation. The relative standard deviation between replicate analyses was less than 4%. The limit of detection expressed as three times the standard deviations of blank (mg/L) were Cd (0.01), Pb (0.005), Ni (0.005), Cr (0.004), Cu (0.003), Co (0.01), Fe (0.45), Mn (0.02), and Zn (0.10). The limits of quantification (mg/L) were Cd (0.01), Pb (0.006), Ni (0.006), Cr (0.005), Cu (0.004), Co (0.01), Fe (0.68), Mn (0.03), and Zn (0.15).The results are expressed as mean ± standard (SD) and one way analysis of variance (ANOVA) was carried out using statistical analysis system (SPSS version 12, Chicago, IL, USA). Differences in concentrations within a given group were tested with ANOVA. Turkey multiple comparison tests were used to compare the differences in concentrations of elements from different drink types.

## Results and Discussion

The summary of mean concentrations for Cd, Pb, Ni, Cr, Cu, Co, Fe, Mn, Zn, Mg, Ca, K, and Na in different types of traditional alcoholic beverages is presented in Table1. The concentrations of metal in the various types showed significant variation at 95% probability level. However, no significant difference was observed in the concentrations of Co in raphia wine and Mg in burukutu. Since relatively few data are currently available on the concentrations of metals in these traditional alcoholic beverages, most comparisons in this study shall be made with metal concentrations found in other alcoholic beverages.

**Table 1:** Concentrations of metals (mg/L) in some traditional alcoholic beverages.

Element	Oil palm wine (Nkwu)^1^ (*n* = 15)					Raphia palm wine (Ogoro)^1^ (*n* = 15)					Burukutu (*n* = 15)					Pito (*n* = 15)					Local gin (Ogogoro) (*n* = 15)				
	Mean	SD	Minimum	Maximum	CV (%)	Mean	SD	Minimum	Maximum	CV (%)	Mean	SD	Minimum	Maximum	CV (%)	Mean	SD	Minimum	Maximum	CV (%)	Mean	SD	Minimum	Maximum	CV (%)
Cd	0.02^3^	0.01	0.01	0.03	26.2	0.05	0.01	0.02	0.07	28.6	0.04	0.01	0.03	0.05	13.9	0.03^3^	0.01	0.02	0.05	21.8	0.02^3^	0.02	0.004	0.07	100
Pb	0.01	0.02	nd	0.04	132.7	0.03	0.01	0.02	0.04	26.9	0.19	0.26	0.07	1.12	133.8	0.07	0.03	0.03	0.15	46.2	0.11	0.06	nd	0.18	54.5
Ni	nd	–	nd	nd	–	nd^2^	–	nd	Nd	–	0.11	0.04	0.06	0.21	35.2	0.002	0.005	nd	0.02	223.5	0.04	0.02	nd	0.08	50
Cr	0.05	0.03	nd	0.08	55.5	nd	–	nd	Nd	–	0.15	0.07	0.09	0.29	48.2	0.02^3^	0.03	nd	0.09	120	0.01^3^	0.01	nd	0.03	100
Cu	0.60	0.26	0.40	1.09	42.7	0.49	0.20	0.05	0.83	41.2	0.49	0.26	0.06	1.19	53.6	0.09	0.09	0.004	0.38	100	0.26	0.32	nd	0.81	123
Co	0.03	0.01	0.01	0.04	31.9	0.06	0.01	0.05	0.08	12.5	0.08	0.01	0.07	0.09	92.1	0.06	0.01	0.02	0.07	22.5	0.01	0.01	nd	0.03	100
Fe	1.21	0.65	0.57	3.10	53.7	1.52	0.62	0.78	2.64	40.4	10.3	3.37	1.72	15.54	32.7	2.59^1^	0.91	1.15	3.77	3.51	0.30	0.31	nd	1.0	103
Mn	0.32	0.52	0.03	1.53	164.2	2.32	1.03	0.44	3.97	44.4	3.97	0.75	2.40	4.96	19.0	0.87	0.62	0.42	2.73	70.9	0.02	0.03	nd	0.04	150
Zn	0.54	0.61	0.15	2.38	113.5	3.86	12.36	0.23	48.50	319.8	3.57	0.89	238	5.79	25.0	1.34	0.49	0.34	2.31	36.7	0.12	0.08	nd	0.29	667
Mg	43.27	15.75	0.33	68.30	36.3	43.20	27.72	24.08	137.72	64.2	301.3	47.58	229.50	375.1	15.7	91.19	29.14	39.99	156.63	31.9	2.08	3.06	0.23	9.56	147
Ca	12.75	5.28	6.98	22.62	41.4	19.09	10.11	4.74	48.77	53.0	49.2	8.57	3.42	64.73	17.4	21.65	39.94	5.29	164.30	184.5	2.21	2.50	nd	7.35	113
K	926.1	290.1	555.3	1353.8	31.3	517.5	142.1	190	707.5	27.5	332.8	122	113.0	442.5	36.7	162.8	156.4	51.3	681.3	96	35.05	65.92	1.69	152.88	188
Na	6.43	1.97	4.0	7.1	30.7	14.98	8.35	3.5	28.75	55.8	59.1	15.8	50.0	112.5	26.8	20.7	6.7	13.8	36.3	33.6	NA	NA	NA	NA	

NA, not analyzed.

1 Igbo language.

2 Not significant at*P* < 0.05 with the same type.

3 Not significant at*P* < 0.05 when compared with other drink types.

The mean concentrations of Cd varied from 0.02 mg/L in oil palm wine and local gin to 0.05 mg/mL in raphia palm wine. The permissible limit of Cd in wine as specified by the International Organization for grapes and wine is set at 0.01 mg/L (OIV 2008). The mean concentrations of Cd in these categories of alcoholic beverages were above the permissible limit. A wide range of Cd concentrations have been reported in alcoholic beverages in the literature which are comparable to levels observed in the present study. For example, Ukhun et al. (2005) reported Cd concentrations ranging from not detected to 0.31 *μ*g/mL for bottled and fresh palm wines in Nigeria. Amidzi Klaric et al. (2011) reported Cd concentrations in the range of 0.55–9.94 *μ*g/L in Croatian blackberry wines. In 2003, the average concentration of Cd in Brazilian beer was reported as 0.0016 mg Cd/L (Soares and Moraes 2003). Cadmium concentrations below the limit of detection have been reported in Ethiopian wines (Woldemariam and Chandravanshi 2011).

The highest mean concentration of Pb was observed in burukutu (0.19 mg/L). The mean concentrations of Pb in the palm wine, burukutu, pito, and local gin were below 0.2 mg/L, the permissible limit for Pb set by OIV (2008). Soares and Moraes (2003) reported lead concentrations ranging from not detected to 0.29 mg Pb/L in Brazilian Beer. Pb concentrations in the range of <0.001–0.047 mg/L have been reported for canned beer in Nigeria (Iwegbue 2010). Donadini et al. (2008) reported that the average concentration of Pb in beers from the Italian market as 1.83 ± 3.24 *μ*g/L. In 2011, the concentrations of Pb in Ethiopian wines were reported as 0.14–0.31 mg/L (Woldemariam and Chandravanshi 2011). The concentrations of Pb found in this study are comparable levels found in brewed beers (Soares and Moraes 2003; Donadini et al. 2008; Iwegbue 2010) However, the Pb concentrations in our samples were lower than levels found in red wines, Brazilian cachacas, and spirits (Nascimento et al. 1999; Pohl 2007; Woldemariam and Chandravanshi 2011).

The concentrations of Ni were below the limit of detection (0.005 mg/L) in all the palm wine samples, and some of the pito and local gin samples examined. The highest mean concentration of Ni was observed in burukutu (0.11 mg/L). The concentrations of Ni in these alcoholic beverages followed the order: burukutu > local gin > pito > palm wine. Nickel has numerous reported mechanisms of toxicity including redox-cycling and inhabitation of DNA repair as well as exhibiting allergenic/sensitizing effects (Hague et al. 2008). The permissible limit for nickel in drinking water is 0.02 mg/L (Standards Organisation of Nigeria (SON) 2007). The mean concentrations of nickel in these alcoholic beverages were below the guideline value for nickel in water except for burukutu and local gin. A wide range of Ni concentrations have been reported in the literature for alcoholic beverages. For example, Anjos et al. (2003) reported Ni concentrations in the range of not detected to 0.3 mg/L in red and white wines from Brazil, Portugal, and Chile. Nascimento et al. (1999) recorded Ni contents of 0.14–0.12 mg/L in Brazilian cachaca and international spirits. Nickel concentrations ranging from 0.04 to 0.1 mg/L have been reported for imported canned beer in Nigeria (Iwegbue 2010). The concentrations of Ni found in our samples are comparable to levels found in alcoholic beverages in the literature.

Chromium toxicity is very dependent on the species and oxidation states present. It is normally found in the considerably less toxic trivalent state in foods and is poorly absorbed in the gastrointestinal tract. Chromium has been reported to have beneficial effects on type II diabetes (Hague et al. 2008). However, the hexavalent form is carcinogenic. It has been estimated that human requires nearly 1 *μ*gCr/day. The mean concentrations of Cr found in these alcoholic beverages ranged from nd to 0.15 mg/L. The highest mean level of Cr was observed in burukutu while the concentrations of Cr in the raphia wine samples examined were less than 0.005 mg/mL. Higher levels of Cr were observed in the fermented products (burukutu and pito) compared with the levels found in the unprocessed palm wine and distilled local gin. The possible sources of Cr in burukutu and pito are due to processing and storage. The permissible limit of Cr in drinking water in Nigeria is set at 0.05 mg/L (Standards Organisation of Nigeria (SON) 2007). The mean concentrations of Cr in these alcoholic drinks were below the limit except for burukutu. The concentrations of Cr found in these alcoholic beverages are comparable to levels of Cr reported for alcoholic drinks in the literature. For example, Nascimento et al. (1999) recorded Cr level of 0.012–0.060 mg/L in Brazilian Cachacas. Chromium contents of 12.86–13.30 mg/L have been reported in Croatian blackberry wines (Amidzi Klaric et al. 2011). In Ethiopia, Cr concentrations in the range of not detected to 0.09 mg/L has been reported for red and white wine (Woldemariam and Chandravanshi 2011). Chromium concentrations of 0.01–0.41 mg/L have been reported for German wines (Pohl 2007). Chromium concentrations in wines from Brazil, Portugal, and Chile were found to be in the range of not detected to 0.11 mg/L (Anjos et al. 2003).

The physiologic roles of Cu include amine oxidases, caeruloplasmin, dopamine hydrolase, and collagen synthesis (Marais and Blackhurst 2009). The mean concentrations of Cu in examined samples ranged from 0.09 to 0.60 mg/L. The highest mean concentration of Cu was observed in oil palm wine while the lowest mean concentration of Cu was observed in pito. The permissible limit of Cu in wine is set at 1 mg/L by the international organization for grapes and wine (OIV, 2008). The mean concentrations of Cu in these alcoholic beverages were below the permissible limit. Copper concentrations in wine may come from the residues of copper-based pesticides in addition to transport from the soil (Sass-Kiss et al. 2008). In 2010, the concentrations of Cu in Serbian wines were found to be in the range of 0.07–0.57 mg/L (Kostic et al. 2010). Copper concentrations ranging from 0.058 to 0.767 mg/L were reported for Croatian blackberry wines (Amidzi Klaric et al. 2011). Iwegbue (2010) reported Cu concentrations of 0.05–0.10 mg/L in canned beer consumed in Nigeria. Ukhun et al. (2005) reported Cu concentrations in bottled and fresh palm wine in Benin City, Nigeria as 1.07–7.52 mg/L. Copper concentrations ranging from not detected to 0.4 mg/L have been reported for wines from Brazil, Chile, and Portugal (Anjos et al. 2003). The concentrations of Cu reported in this study were similar to Cu levels found in wine, brewed beers, and spirits in the literature but were far lower than the range reported by Ukhun et al. (2005).

The concentrations of Co in these alcoholic drinks were in the range of 0.01–0.08 *μ*g/mL. The highest mean concentration of Co was observed in burukutu while the lowest mean concentration of Co was observed in the local gin. Cobalt is an essential trace element and a component of the vitamin B_12_. In 2010, Co concentrations of 0.07–0.1 mg/L were recorded in canned beer consumed in Nigeria (Iwegbue 2010). In Ethiopia, Co concentrations ranging from not detected to 0.09 mg/L were found in wines (Woldemariam and Chandravanshi 2011). Cobalt concentrations in Croatian blackberry wines were found to be in the range of 5.77–7.49 mg/L (Amidzi Klaric et al. 2011). The concentrations of Co recorded in this study were similar to concentrations of Co reported in wines in the literature (Galani-Nikolakaki et al. 2002; Sauvage et al. 2002; Marengo and Aceto 2003; Álvarez et al. 2007; Pohl 2007; Sass-Kiss et al. 2008).

The mean concentrations of Fe ranged from 0.3 to 10.3 mg/L. Burukutu contained higher concentrations of Fe than palm wine, pito, and ogogoro. The permissible limit of Fe in drinking water in Nigeria is set at 0.3 mg/L (Standards Organisation of Nigeria (SON) 2007). The concentrations of Fe in these drinks were higher than the permissible limits for Fe in drinking water except for the local gin (ogogoro). A wide concentration range of Fe have been reported in the literature for wines (Sauvage et al. 2002; Marengo and Aceto 2003; Álvarez et al. 2007; Pohl 2007; La Torre et al. 2008). For example, Kostic et al. (2010) reported Fe concentrations of Serbian wines as 2.93–36.2 mg/L. The concentrations of Fe in Ethiopian wines ranged from 1.42 to 3.16 mg/L (Woldemariam and Chandravanshi 2011). The concentrations of Fe reported in the present study were similar to the concentrations of Fe reported for alcoholic beverages in the literature. Iron concentrations of wines depend on several factors, mostly on the soil, dust, and contamination during harvesting, transportation, and processing. At Fe concentrations greater than 10 mg/L, Fe (III) creates insoluble suspension with tannin and phosphates which are known as hazes or cases (Paleologos et al., 2002). High Fe concentrations in the range of 7–10 mg/L in wines may cause cloudiness or color change.

Manganese serves as an active constituent of several enzymes including antioxidants such as mitochondrial superoxide dismutase among others (Batista et al. 2011). The concentrations of Mn in these alcoholic beverages varied from 0.02 to 3.97 mg/L. Higher concentration range of Mn were recorded in the burukutu samples which varied over very small range as compared with other alcoholic beverages studied. The permissible limit for Mn in drinking water is 0.2 mg/L (Standards Organisation of Nigeria (SON) 2007). The concentrations of Mn in these alcoholic beverages were higher than the permissible level of Mn in water except for the ogogoro samples. The concentrations of Mn found in the present study are similar to levels reported in the literature for wines (Li and Hardy 1999; Sauvage et al. 2002; Kment et al. 2005; Álvarez et al. 2007; Pohl 2007; Sass-Kiss et al. 2008; Woldemariam and Chandravanshi 2011).

The concentrations of Zn in these samples varied from 0.12 mg/L in ogogoro to 3.86 mg/L in raphia palm wine. Burukutu and raphia palm wine generally contained higher concentrations of Zn than the oil palm wine, pito, and ogogoro. The permissible limit of Zn in alcohol is set at 5.0 mg/L (OIV 2008). The concentrations of Zn recorded in this study were below the permissible limit for Zn in wines. In 2005, the concentrations of Zn in fresh and bottled palm wine in Benin City, Nigeria were found to be in the range of 0.98–8.88 mg/L (Ukhun et al. 2005). The low concentrations of Zn found in the samples of ogogoro (local gin) were similar to 0.126–0.137 mg/L range that was reported for Brazilian cachaca (sugar-cane spirit). Zinc concentrations in the range of 0.2–1.3 mg/L have been reported in wines from Brazil, Portugal, and Chile (Anjos et al. 2003). Woldemariam and Chandravanshi (2011) recorded Zn concentrations in the range of 1.82–2.70 mg/L in Ethiopian wines. The concentrations of Zn reported in this study were similar to concentrations of Zn reported in the literatures for wines.

Magnesium deficiency in organisms may lead to serious biochemical and symptomatic changes. This element is involved in more than 300 essential metabolic reactions (Batista et al. 2011). The concentrations of Mg varied considerably according to the type of the alcoholic beverage analyzed. The lowest mean concentration of Mn was observed in ogogoro (2.08 mg/L) while the highest mean concentration was observed in burukutu (301.33 mg/L). The mean concentrations of Mg in the oil palm and raphia palm wines were similar. Magnesium contents in the range of 11.4–97.5 mg/L have been reported in alcoholic beverages and by-products from Spain (Navarro et al. 2007). The concentrations of magnesium recorded in these alcoholic beverages were similar to those found in other types of alcoholic beverages in the literature (Lazos and Alexakis 1989; Li and Hardy 1999; Rebolo et al. 2000; Frias et al. 2002; Sauvage et al. 2002; Marengo and Aceto 2003; Álvarez et al. 2007; Galani-Nikolakaki et al., 2002; Pohl 2007; La Torre et al. 2008; Sass-Kiss et al. 2008). The results of the present study suggest that burukutu is a good dietary source of magnesium.

The mean concentrations of Ca in these alcoholic beverages ranged from 2.21 to 49.23 mg/L. The highest mean concentration of Ca was observed in burukutu, while the lowest mean concentration was observed in the ogogoro. The concentrations of Ca in these drink types varied considerably (*P *<* *0.05). Navarro et al. (2007) reported Ca concentrations in the range of 65.7–185.2 mg/L in alcoholic beverage and by-products from Spain. In Croatia, Ca contents of 101–188.4 mg/L were reported for blackberry wines (Amidzi Klaric et al. 2011). Anjos et al. (2003) reported that the Ca concentrations in wines from Brazil, Chile, and Portugal ranged from 55.1 to 102.9 mg/L. Calcium concentrations of 58–280 mg/L have been recorded for German wines (Pohl 2007). The levels of Ca in French wines were recorded as 65–161 mg/L while Ca concentrations of contents of 17–94 mg/L were found in American wines (Li and Hardy, 1999). In 2011, Ca concentrations ranging from 28 to 37 mg/L were recorded in Ethiopian wines (Woldemariam and Chandravanshi 2011). The concentrations of Ca reported in this study were comparable to values reported for American and Ethiopian wines.

It is clear that potassium was the major mineral in these alcoholic drinks. Potassium concentrations in these alcoholic drinks varied over a wide range. The concentrations of K ranged between 21.65 and 926.1 mg/L. The highest mean concentration of K was observed in oil palm wine while the lowest mean concentration was observed in pito. The distribution pattern of K in these drinks followed the order: oil palm wine > raphia palm wine > burukutu > ogogoro > Pito. In 2011, K concentrations of 924–1507 mg/L have been reported in Croatian blackberry wines (Amidzi Klaric et al. 2011). Potassium concentrations of 462–1147 mg/L were reported for American wines (Li and Hardy, 1999). In Ethiopia, K concentrations of 694–767 mg/L were recorded for wines (Woldemariam and Chandravanshi 2011). Aside from oil palm and raphia wines, the concentration range of K found in the other drink types was lower than the ranges reported by these researchers. The mean concentrations of K in the ogogoro samples were higher than that of the mean levels reported in Brazilian cachacas (Nascimento et al. 1999).

The concentrations of Na in these traditional alcoholic beverages ranged from 4.0 to 7.1 mg/L, 3.5 to 28.75 mg/L, 15.8 to 12.5 mg/L, 13.8 to 36.4 mg/L for oil palm wine, raphia palm wine, burukutu, and pito, respectively. The highest mean concentration of Na was observed in oil palm wine. The OIV acceptable limit of Na in wine is set at 60 mg/L (OIV 2008). The mean concentrations of Na found in these alcoholic beverages were below the permissible limit for Na in wines. However, only 2 samples of burukutu had Na concentrations above the OIV limit. In Croatian blackberry wines, Na concentrations of 11.80–120.10 mg/L were recorded (Amidzi Klaric et al. 2011). In Ethiopia, Na concentrations of 24–25 mg/L were observed in wine (Woldemariam and Chandravanshi 2011). Sodium concentrations of 18.6–81.1 mg/L have been reported for Hungarian wines (Pohl 2007; Sass-Kiss et al. 2008). The concentrations of Na in this study are in conformity with the wine data reported in the literature (Li and Hardy 1999; Cabera-vique et al. 1997; Frias et al. 2002; Sauvage et al. 2002; Marengo and Aceto 2003; Kment et al. 2005; Álvarez et al. 2007; Pohl 2007; Sass-Kiss et al. 2008).

Generally, the concentrations of metals in the distilled product (ogogoro) were lower than that of fermented products (burukutu and pito) and freshly consumed products (palm wine). This is due to the fact that volatilization process favors the transfer of alcohol, other aromatics, and lower molecular weight compounds through the condensation coils and tends to leave behind the heavier metals. Conversely, for fermentation products, metal transfer from the raw materials is largely conserved (Navarro et al. 2007).

### Estimated dietary intake of metals and target hazard quotients

The estimated daily intakes of the studied metals in*μ*g/kg bw/day are provided in Table2. The daily intake of Cd from the consumption of these alcoholic drinks spanned between 0.003 and 0.21 *μ*g/kg bw/day. Higher intakes of Cd were obtained from consumption of raphia wine and burukutu. The tolerable intake value of Cd is set at 1 *μ*g/kg bw/day (World Health Organization (WHO) 1993). The estimated intake value of Cd from consumption of these beverages constituted approximately 0.3–21% of the tolerable intake value.

**Table 2:** Estimated daily intake in*μ*g/kg bw/day based in 250 mL per day.

Element	Oil palm wine	Raphia wine	Burukutu	Pito	Local gin^1^
Cd	0.08	0.21	0.21	0.13	0.003
Pb	0.04	0.13	0.79	0.29	0.02
Ni	0.002^1^	0.002^1^	0.46	0.008	0.007
Cr	0.21	0.002	0.63	0.08	0.002
Cu	2.50	2.04	2.04	0.38	0.04
Co	0.13	0.25	0.33	0.25	0.002
Fe	5.04	6.33	42.9	10.8	0.05
Mn	1.33	9.67	16.5	3.63	0.003
Zn	2.25	16.08	14.9	5.58	0.02
Mg	180.7	180	12.55.5	380	0.35
Ca	53.1	79.5	204.2	90.2	0.38
K	38.59	2156.3	1386.5	678.3	0.06
Na	26.79	59.38	246.3	86.3	–

1 Based on consumption of one shot (10 mL) per day.

The estimated daily intake value of Pb from this study ranged from 0.02 to 0.79 *μ*g/kg bw/day. The tolerable daily intake of Pb is set at 3.6 *μ*g/kg bw/day. The estimated intake values of Pb in this study constitute approximately 0.5–21.9% of tolerable intake value. The highest intake value of Pb is obtained from consumption of burukutu.

The tolerable daily intake of Ni is 5 *μ*g/kg bw/day. The estimated daily intake of Ni ranged from 0.002 to 0.46 *μ*g/kg bw/day. The intake values of Ni obtained from this study constituted 0.04–9.8% of tolerable daily intake values. In this study, the highest dietary intake of Cr was obtained from the consumption of burukutu (0.63 *μ*g/kg bw/day) while lower intakes of Cr were obtained from consumption of raphia wine and ogogoro (0.002 *μ*g/kg bw/day). The recommended dietary allowance of Cr is 130 *μ*g/day/person which is equivalent to 2.2 *μ*g/kg bw/day. The intakes of Cr from the consumption of these drinks were below the recommended dietary allowance value. The dietary intakes of Co from consumption of any of these alcoholic beverages varied from 0.002 to 0.33 *μ*g/kg bw/day. The intake values for Co in raphia wine and pito were similar. The highest intake value of Co was obtained from consumption of burukutu in this study. The normal daily intake of Co is reported to be in the range of 2.5–3.0 *μ*g/day. Poisoning occurs within a range greater than 20–30 mg Co per day (Hokin et al. 2004). The recommended dietary allowance for Co is 100 *μ*g/day (Reilly 2004; Nutrition Data 2008). The estimated dietary intakes of Co in this study were below the recommended daily intake value.

The recommended dietary allowance (RDA) for Cu ranged from 900 *μ*g to 30 mg/day person which is equivalent to 15–500 *μ*g/kg bw/day (World Health Organization (WHO) 1993). The intake value for Cu in this study ranged from 0.04 to 2.50 *μ*g/kg bw/day. The highest intake value of Cu was obtained from the consumption of oil palm wine than that of the other drink types. The estimated intake values for Cu constituted 0.008–0.5% of the upper limit of recommended dietary allowance value.

The Joint FAO/WHO Export Committee on Food Additives (JECFA) provisional maximal tolerable daily intake of Zn is 1000 *μ*g/kg bw/day (World Health Organization (WHO) 1982). The EVM safe upper limit (SUL) for Zn is 4.2 mg/day (equivalent to 700 *μ*g/kg bw/day in a 60 kg adult) for total dietary intake (EVM 2003). The estimated intakes of Zn from the consumption of these alcoholic beverages ranged from 0.02 to 16.08 *μ*g/kg bw/day. The highest intake value was obtained from consumption of raphia wine. The estimated intake values of Zn in this study were less than 1.7% of the provisional maximal tolerable daily intake of Zn.

The recommended dietary allowance values for Fe and Mn were 10–18 mg/day person and 2–5 mg/day person, respectively. The estimated intake of Fe and Mn from consumption of these drink types varied from 0.05 to 42.9 *μ*g/kg bw/day and 0.003–16.5 *μ*g/kg bw/day for Fe and Mn, respectively. The intake values of Fe and Mn in this study were less than 1% of the recommended dietary allowance value for Fe and Mn.

The RDA for Mg for male and female healthy adults are 400–420 and 310–320 mg mg/day respectively (Institute of Medicine, 2002) while the recommended dietary allowance value for Ca is set at 1000 mg Ca/day^1^ (Institute of Medicine, 2010). The estimated intake values of Mg and Ca varied considerably among these alcoholic beverages. The highest dietary intakes of Mg and Ca were obtained from the consumption of Burukutu (Table2). The estimated intake values of Mg and Ca in this study were less than 0.5% of the recommended dietary allowance values.

The daily intake of K and Na in this study ranged from 0.06 to 38.59 *μ*g/kg bw/day and 26.79 to 246.3 *μ*g/kg bw/day, respectively. The highest intake value for K was obtained for consumption of 250 mL of palm wine per day while the highest intake of Na was observed for consumption of burukutu.

### Estimation of target hazard quotients

To assess the level of concern arising from metal concentration, THQ values were calculated using the measured metal concentration in the intact samples for nine potentially toxic metals in these alcoholic beverages. The THQ is a ratio between the measured concentrations and the oral reference dose, weighted by the length and frequency of exposure, amount ingested, and body weight (Hague et al. 2008).

The THQ is calculated by the formula established by the Environmental Protection Agency (USEPA 1989) using equation (1).


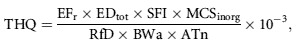
(1)

where EF_r_ is the exposure frequency (days/year); ED_tot_ is exposure duration (year): SFI is the mass of selected dietary ingested (g/day); MCS_inorg_ is the concentration of inorganic species in the dietary components (mg/kg wet weight); RfD, oral reference dose (mg/kg/day); BWa; the average adult body weight; ATn; averaging time for noncarcinogens (Day); and 10^−3^; unit conversion factor. The oral reference doses in mg kg^−1^ day^−1^ Cd (1 × 10^−3^), Pb (1.5), Ni (2.0 × 10^−3^), Cr (1.5), Cu (4.0 × 10^−2^), Co (3.0 × 10^−4^), Fe (7.0 × 10^−1^), Mn (1.4 × 10^−1^), and Zn (3.0 × 10^−1^). For estimation of dietary intakes of metals, ingestion rate of 250 mL/day of palm wine, burukutu, and pito was assumed. For ogogoro, daily intake calculation was based on per capital consumption of 3.6 L of pure alcohol. For this study, the length of exposure was set to 12191 days for Nigeria based on the average life expectancy of 48.4 years, from 15 years of age (World Health Organization (WHO) 2004). The average weight of 60 kg adult was adopted in this study.

The estimated THQ values (Table3) for individual metals from consumption of one 250 mL glass per day was less than 1 except for cobalt in burukutu (THQ = 1.1). The THQ values <1 indicate safe level while THQ values >1 indicate levels of concerns. THQ values are additives not multiplicative, thus a THQ value of 20 is larger but not 10-fold greater than a THQ = 2. The combined THQ values for all metals examined were ≥1 <2 for raphia wine, burukutu, and pito, while the combined THQ values for oil palm wine and that of ogogoro were <1. From the estimated THQ values, no lifelong health concern of metals is associated with the consumption of these alcoholic beverages.

**Table 3:** Estimated target hazard quotient (THQ) from consumption of traditional alcoholic beverages in Nigeria.

Element	OPW	RPW	BRK	PIT	OG
Cd	0.08	0.21	0.21	0.13	0.003
Pb	0.00	0.00	0.00	0.00	0.00
Ni	0.00	0.00	0.00	0.00	0.00
Cr	0.00	0.00	0.00	0.00	0.00
Cu	0.06	0.05	0.05	0.01	0.01
Co	0.43	0.83	1.10	0.83	0.002
Fe	0.01	0.01	0.06	0.02	0.00
Mn	0.01	0.07	0.12	0.01	0.00
Zn	0.01	0.06	0.05	0.02	0.00
Combined THQ	0.6	1.23	1.61	1.00	0.05

Opw, Oil palm wine; PRW, Raphia palm wine; BRK, Burukutu; OG, Ogogoro (local gin).

## Conclusions

The results of the present study indicated that the metals occurred in these alcoholic beverages at concentrations below international statutory limits permissible in wines and spirits except for Cd, and were similar to concentrations of metals found in other alcoholic beverages in the literature. The estimated dietary intakes of metals from the consumption of these alcoholic beverages were less than 2% recommended dietary allowance values except for Cd and Pb whose estimated maxima dietary intakes were up to 23% of provisional tolerable daily intake. From the estimated target hazard values, no lifelong health concerns of metals are associated with the consumption of these alcoholic beverages. However, excessive consumption of these drinks has severe health implications based on its alcohol contents and could also lead to very high intakes of Cd and Pb. The results of this study indicated that the unprocessed freshly consumed products and fermented products contained higher metal loads compared with the distilled product (ogogoro). Burukutu contained the higher concentrations of most elements in comparison with the other products which implied that persons who take burukutu in preference to the other traditional drinks are likely to be more exposure to metals.

## Conflict of Interest

None declared.

## References

[b1] Álvarez M, Moreno IM, Jos A, Cameán AM, González AG (2007). Differentiation of two Andalusian DO “fino” wines according to their metal content from ICP-OES by using supervised pattern recognition methods. Microchem. J.

[b2] Amidzi Klaric D, Klaric I, Velic D, Vedrina Dragojevic I (2011). Evaluation of mineral and metal contents in Croatian Blackberry wines. Czech J. Food Sci.

[b3] Anjos MJ, Lopes RT, de Jesus EFO, Moreira S, Barroso RC, Castro CRF (2003). Trace elements determination in red and white wines using total reflection x-ray fluorescence. Spectrochemi. Acta Part B.

[b4] Batista BL, De Oliveira Sauza VC, Da Silva FG, Barbosa F (2011). Survey of 13 trace elements of toxic and nutritional significance in rice from Brazil and exposure assessment. Food Addit. Contam. Part B.

[b5] Cabera-vique C, Teissendre PL, Cabanis MT, Cabanis JC (1997). Determination and levels of chromium in French wine and grapes by graphite furnace atomic absorption spectroscopy. J. Agric. Food Chem.

[b8] Expert Group on Vitamins and Minerals (evm) (2003). http://www.food.gov.uk/multimedia/pdfs/vitmin2003.pdf.

[b9] Ezeagu IE, Fafunso MA, Ejezie FE (2003). Biochemical constituents of palm wine. Ecol. Food Nutr.

[b10] FAO (1998).

[b11] Frias S, Conde JE, Rodriguez MA, Dohual V, Perez-Trujillo JP (2002). Metallic content of wines from the Canary Island (Spain). Application of artificial neural network to data analysis. Nehrung.

[b12] Galani-Nikolakaki S, Kallithrakas-Kontos N, Katsanos AA (2002). Trace element analysis in Cretan wines and wine products. Sci. Total Environ.

[b13] Hague T, Petroczi A, Andrews PLR, Barker J, Naughton DP (2008). Determination of metal ion contents of beverages and estimation of target hazard quotients: a comparative study. Chem. Cent. J.

[b45] Hokin B, Adams M, Ashton J, Louir H (2004). Analysis of cobalt content in Australian foods. Asia Pac. J. Clin. Nutr.

[b46] Institute of Medicine (2002).

[b14] Iwegbue CMA (2010). Composition and daily intakes of some trace metals from canned beers in Nigeria. J. Inst. Brew.

[b15] Kment P, Mihaljevic M, Ettler V, Sebek O, Strnad I, Rohlova I (2005). Differentiation of czech wines using multielement composition – a comparison with vineyard soil. Food Chem.

[b16] Kostic D, Mitic S, Miletic G, Despotovic S, Zarubica A (2010). The concentrations of Fe, Cu and Zn in selected wines from South-East Serbia. J. Serb. Chem. Soc.

[b17] La Torre GK, La Pera L, Rando R, Lo Turco V, Di Bella G, Saitta M (2008). Classification of Marsala wines according to their polyphenols, carbohydrate and heavy metals. Food Chem.

[b18] Lazos ES, Alexakis A (1989). Metal content of some Greek wines. Int. J. Food Sci. Technol.

[b19] Li P, Hardy JK (1999). Characterization of Ohio wines using multivariate analysis. J. Wine Res.

[b20] Marais AD, Blackhurst DM (2009). Do heavy metal counter thee potential health benefits of wine?. JEMDSA.

[b21] Marengo E, Aceto M (2003). Statistical investigation of the differences in the distribution of metals in Nebbiolo- based wines. Food Chem.

[b22] Mihaela A, Dumitrescu V, Tânase IGH, Maria P, Nedelcu R (2009). The optimization of the methods for Cu, Zn and pb content determination in Romanian wines by AAS after dry or microwave mineralization. Rom. Biotechnol. Lett.

[b23] Nascimento RF, Bezerra CWB, Furuya SMB, Schultz MS, Polastro LR, Lima Neto BS (1999). Mineral profile of Brazilian cachacas and other international spirits. J. Food Compos. Anal.

[b24] Naughton DP, Petroczi A (2008a). Heavy metal ions in wine: metal- analysis of target hazard quotients reveal health risks. Cent. Chem. J.

[b25] Naughton DP, Petroczi A (2008b). The metals ion theory of again aging, dietary target hazard quotient beyond radicals’. Immun. Ageing.

[b26] Navarro M, Velasco C, Jodrab A, Terre SC, Olava M, Lopez H (2007). Cu, Zn, Ca and Mg content of alcoholic beverages and by-products from Spain: nutritional supply. Food Addit. Contam.

[b27] Nutrition Data (2008). http://www.nutritiondata.com.

[b28] OIV-Organisation Internationale de la Vigue et du VIN (2008).

[b47] Paleologos EK, Giokas DL, Tzouwara-Karayanni SM, Karayannis MI (2002). Micelle mediated methodology for the determination of free and bound iron in wines by flame atomic absorption spectrometry. Anal. Chem. Acta.

[b29] Pohl P (2007). What do metals tell us about wine?. Trends Anal. Chem.

[b30] Power KM, Smith-Weller T, Franklin GM, longstreth WT, Swanson PD, Checkoway H (2003). Parkinson's disease risk associated with dietary iron, manganese, and other nutrient intakes. Neurology.

[b31] Rebolo S, Pena RM, Latrorre MJ, Garcial S, Botana AM, Herrero C (2000). Characterization of Galicia (Nw Spain) Ribeira Sacra wines using pattern recognition analysis. Anal. Chem. Acta.

[b32] Reilly C (2004). The nutritional trace metals.

[b34] Sass-Kiss A, Kiss J, Havadi B, Adanyi N (2008). Multivariate statistical analysis of botrytised wines of different origin. Food Chem.

[b35] Sauvage L, Frank D, Stearne J, Millikan MB (2002). Trace metals studies of selected white wines: an alternative approach. Anal. Chim. Acta.

[b36] Soares LMV, De Moraes AMM (2003). Lead and content of Brazilian beers. Cienic. Tecnol. Aliment. Campinas.

[b37] Standards Organisation of Nigeria (SON) (2007).

[b38] Ukhun ME, Okolie NP, Onyerinde AO (2005). Some mineral profile of fresh and bottled palm wine – a comparative study. Afr. J. Biotechnol.

[b39] USEPA (1989).

[b40] Valko M, Rhodes CJ, Moncol J, Zakovic M, Mazur M (2006). Free radicals, metals and anti-oxidant in oxidative stress-induced cancer. Chem. Biol. Interact.

[b41] Woldemariam DM, Chandravanshi BS (2011). Concentration levels of essential and non-essential elements in selected Ethiopian wines. Bull. Chem. Soc. Ethiopia.

[b42] World Health Organization (WHO) (1982). Safety evaluation of certain food additives and contaminants: Zinc, WHO additives series No 17.

[b43] World Health Organization (WHO) (1993).

[b44] World Health Organization (WHO) (2004). Global status report on alcohol.

